# The extrachromosomal circular DNA atlas of aged and young mouse brains

**DOI:** 10.1038/s41597-024-03146-x

**Published:** 2024-03-27

**Authors:** Xiaoning Hong, Jing Li, Peng Han, Shaofu Li, Jiaying Yu, Haoran Zhang, Jiang Li, Yonghui Dang, Xi Xiang

**Affiliations:** 1 Scientific Research Center, The Seventh Affiliated Hospital of Sun Yat-Sen University, Shenzhen, Guangdong China; 2 College of Medicine and Forensics, Xi’an Jiaotong University Health Science Center, Xi’an, Shanxi China; 3 Department of Biology, University of Copenhagen, Copenhagen, Denmark

**Keywords:** Transcriptional regulatory elements, DNA sequencing

## Abstract

Extrachromosomal circular DNA (eccDNA) refers to a distinct class of circular DNA molecules that exist independently from linear chromosomal DNA. Extensive evidence has firmly established the significant involvement of eccDNA in cancer initiation, progression, and evolutionary processes. However, the relationship between eccDNA and brain aging remains elusive. Here, we employed extrachromosomal circular DNA sequencing (Circle-seq) to generate a comprehensive dataset of eccDNA from six brain structures of both young and naturally-aged mice, including the olfactory bulb, medial prefrontal cortex, nucleus accumbens, caudate putamen, hippocampus, and cerebellum. Furthermore, through database annotation, we characterized the properties of mouse brain eccDNA, thereby gaining insights into the potential functions of eccDNA in the mouse brain. In conclusion, our study addresses a previously unexplored area by providing a comprehensive molecular characterization of eccDNA in brain tissues. The data presented in the study can be used as a fundamental resource to associate the molecular phenotypes of eccDNA with brain aging and gain deep insights into the biological role of eccDNA in mammalian brain aging.

## Background & Summary

Aging is a complex process that leads to a decline in organ function and life quality as time passes^1^. During the aging process, changes occur in the brain, including shrinkage of brain size, remodelling of vascular structure and poor cognition^2^. At the histological level, this is manifested by decreased brain weight and atrophy, white matter atrophy occurring later than gray matter, ventricular enlargement, cerebral vascular changes, and reduced neuronal repair capacity^3^. In daily life, this is reflected in a decline in memory, learning ability, sensory perception and motor coordination^4^. This process is characterized by its irreversibility, progressive nature and cumulative effects^5^. These changes can serve as markers of brain aging and contribute to the development of neurodegenerative diseases such as Alzheimer’s disease, Parkinson’s disease, temporal lobe dementia and stroke^4^.

Extrachromosomal circular DNA (eccDNA) refers to circular DNA molecules that originate from chromosomal DNA but exist independently in the cell nucleus and range in size from hundreds to millions of base pairs. eccDNA was first observed in the boar sperm by Alix Bassel and Yasuo Hoota in 1965 through electron microscopy^6^. There is increasing evidence indicating that eccDNAs are widely present in eukaryotic organisms, including plants, animals, and fungi, and they play important roles in various biological processes^7–9^, including gene amplification^10^, regulating RNA expression^11^ and senescence^12^. Some studies also suggest that eccDNA may be involved in sequestering transcription factors, releasing molecules for intercellular communication and stimulating the innate immune pathways^13^. Recently, scientists have paid more attention to the role of eccDNA in tumor initiation and malignant progression, particularly the genetic heterogeneity among tumor cells caused by the amplification of oncogenes and drug resistance genes^14^. Despite its vital role in biology, relatively little is known about its distribution, function and clinical impact on brain aging. Ain Q *et al*. proposed that eccDNA plays a key role in aging and neurodegeneration of the central nervous system, but there is currently a lack of systematic studies in this area^15^. In the present study, we described the genome atlas of eccDNA from various brain structures in mice with aging brains for the first time.

**Fig. 1:**
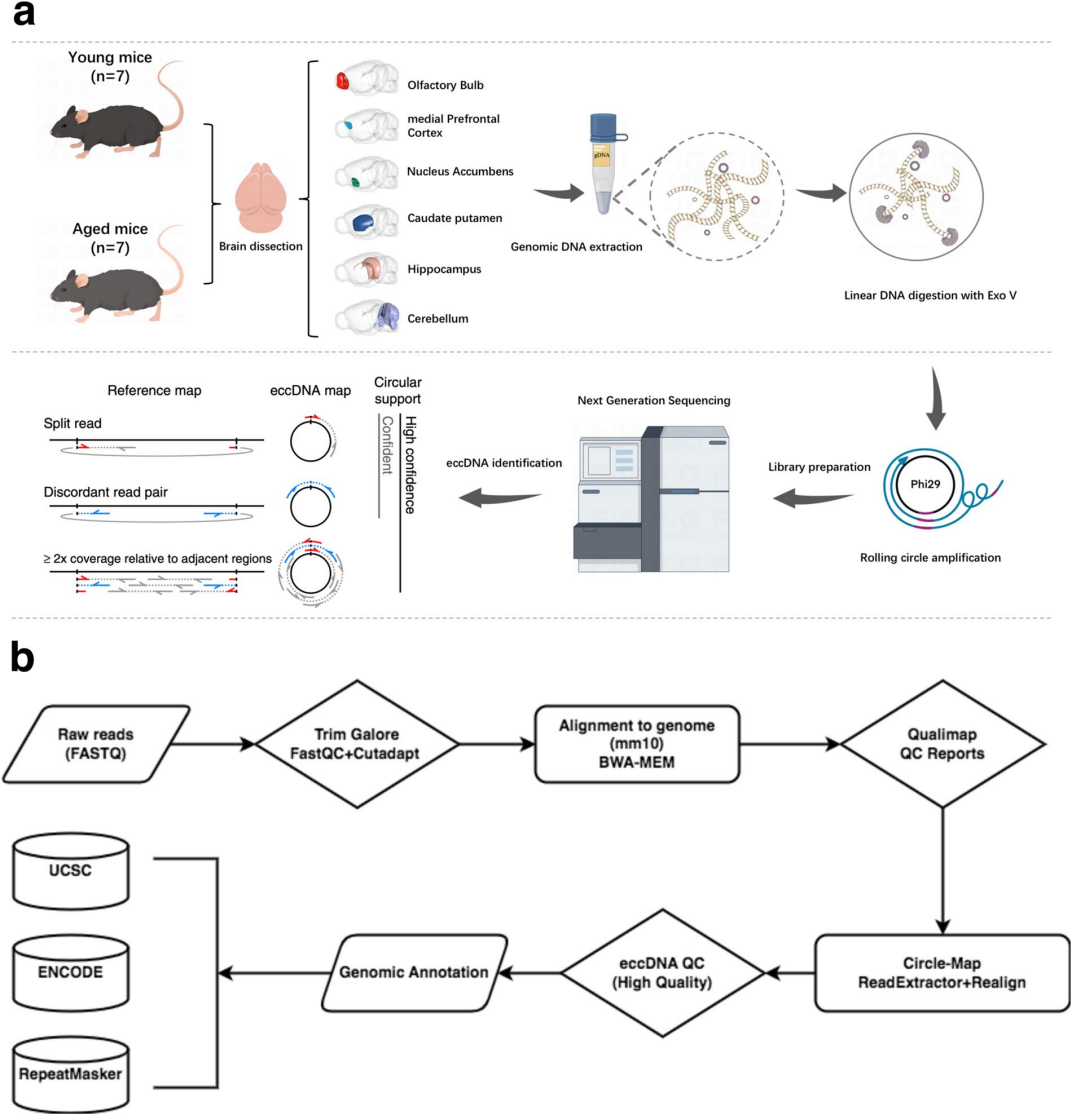
Schematic overview of the experimental design and data analysis. (**a**) Workflow illustrating the process of mouse brain eccDNA purification and identification. (**b**) Bioinformatics pipeline for analysing mouse brain eccDNA.

**Fig. 2:**
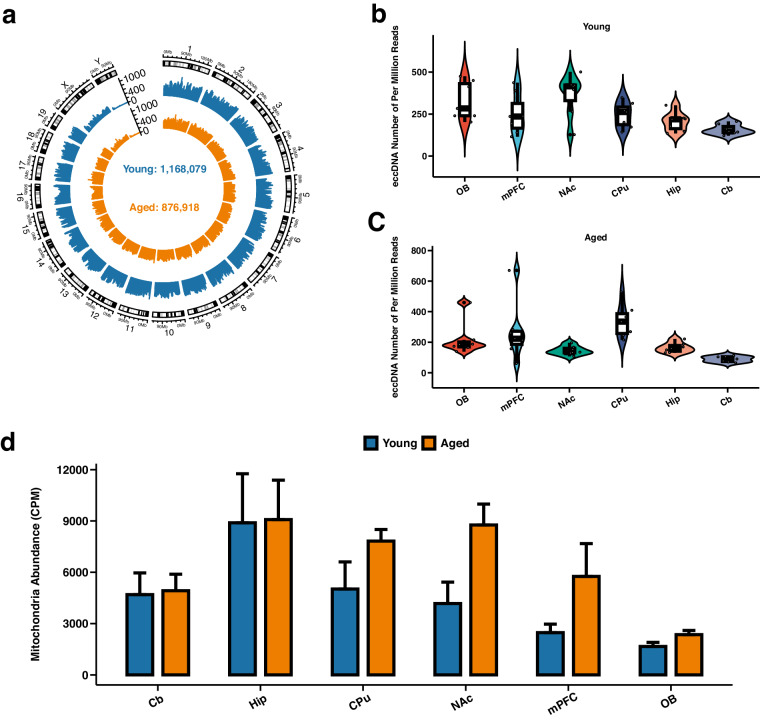
EccDNA characteristics in the mouse brain. (**a**) Circos plot demonstrating the genome-wide distribution of eccDNA in both the Aged and Young groups. (**b,c**) Violin plots demonstrating the distribution of eccDNA per million mapped reads (EPM) across six brain structures for both groups. (**d**) Bar plot demonstrating the mitochondrial abundance in both the Aged and Young groups. The quantification was performed using the split-reads CPM (counts per million) normalization method.

In this Data Descriptor, we conducted rigorous quality control (QC) measures to ensure the high quality of the Circle-seq data. The visualization summary of the study design and workflow is presented in Fig. 1a. The data analyses were performed using a standard pipeline (Fig. 1b). We established seven biological replicates for Circle-seq in each mouse brain structure, where seven young and aged mice were involved and eccDNA data were collected to confirm the authenticity of the datasets. The results showed that in total 876,918 and 1,168,079 eccDNAs were identified in the aged and young group, respectively (Fig. 2a). On average, the number of eccDNAs per brain structure in the aged group was 20,879 (ranging from 5,662 to 75,197), while in the young group it was 27,811 (ranging from 9,819 to 51,781) (Supplementary 1). After normalization, the average eccDNA count per million mapped reads (EPM) in the aged group was 203 (ranging from 89 to 338) per brain structure, compared to 258 (ranging from 158 to 364) in the young group (Fig. 2b,c and Supplementary Table 1). In addition, mitochondrial DNA plays a vital role as a positive control in the detection of eccDNA. The ratio of mitochondrial reads in the young and the aged group was 11.62% and 11.08% (mtDNA read counts divided by all the raw read counts), respectively. The abundance of mitochondrial DNA was quantified using the split-read counts per million reads (CPM) normalization method. The mean CPM values of mitochondrial DNA in the young and aged groups were 4486 and 6450, respectively (Fig. 2d). It indicated that the mitochondrial content of the samples was 0.45% and 0.65% (mtDNA split-read counts divided by the eccDNA split-read counts in each group), respectively.

**Fig. 3:**
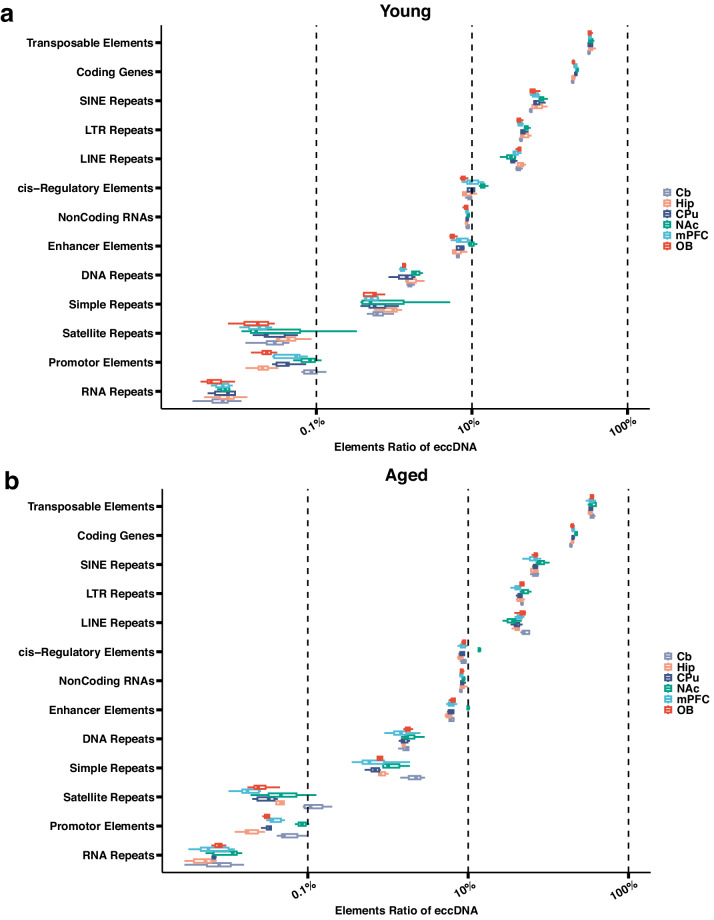
Genomic annotation of mouse brain eccDNA. (**a,b**) The ratio of genomic element from eccDNA annotation across six brain structures for both the Aged and Young groups.

The functional annotation results indicate that the most commonly eccDNA-carried elements are transposable elements, coding genes and cis-regulatory elements, accounting for 58.70%, 52.23% and 15% respectively in the aged group. In the young group, these elements account for 60%, 40%, and 20% respectively (Fig. 3c,d). In addition to that, the detected eccDNAs also contain considerable amounts of repetitive elements, including the SINE, LTR and LINE repeats (Fig. 3c,d).

We provided annotated bed files for each sample, which contain the location information of eccDNAs for each brain structure. These data not only can be a valuable resource for investigating the molecular phenotype association between eccDNA and brain aging, but also contribute to the research in pathogenesis and therapeutics of neurodegenerative diseases.

## Methods

### Experimental animal and samplings

The animals used in this study were approved by the Biomedical Ethics Committee of the Medical School of Xi’an Jiaotong University (No:2017-648). In this study, a total of seven male mice at 3 months of age (equivalent to ~20 years in humans) were collected for the young group, and seven male mice at 19 months of age (equivalent to ~60 years in humans) were collected for the aged group^16^. The mice were euthanized using the cervical dislocation method, and six brain structures, including the olfactory bulb (OB), medial prefrontal cortex (mPFC), nucleus accumbens (NAc), caudate putamen (CPu), hippocampus (Hip) and cerebellum (Cb), were isolated.

### eccDNA isolation and purification from brain tissue

Following the protocol of our previous study, we isolated and amplified eccDNA from mouse brain tissue samples^17^. Briefly, total genomic DNA was extracted from each tissue sample (~ 10 mg) by the MagAttract HMW DNA Kit (QIAGEN, Germany) according to the manufacturer’s instruction. 100 ~ 1000 ng genomic DNA of each sample (depending on the tissue size and the resulting DNA amount. For example, the mouse mPFC tissue was very small and the input DNA amount was ranging from 129 ng to 1000 ng in this study) was treated with plasmid-safe ATP-dependent DNase (PSD) (Lucigen) to remove linear DNA. A 50 μL reaction mixture consisting of 400 ~ 1000 ng genomic DNA, 2 μL PSD, 5 μL 10 X PSD Buffer, 5 μL ATP solution (25 mM), and ddH2O was carried out at 37°C continuously for 7–12 days. Additional ATP solution and PSD (10 μL supplementary system containing 2 μL 10 X PSD Buffer, 2 μL ATP solution (25 mM), 2 μL PSD and 4 μL ddH2O) was added into the reaction mixture every 24 hours. Agarose gel electrophoresis was conducted on DNA samples both before and after PSD digestion to confirm the thorough elimination of linear DNA in each sample. Only the PSD-digested products lacking visible DNA bands were used for the subsequent rolling circle amplification (RCA) reaction. After gel test and before RCA, the PSD-digested mixture was inactivated at 70°C for 30 minutes and purified using VAHTS DNA Clean Beads (Vazyme). The resulting eccDNA product was eluted in 30 μL nuclease-free water and stored in −80°C refrigerator or subjected to the subsequent RCA reaction.

### Rolling circle amplification of eccDNA

The purified eccDNA was subjected to RCA using the Phi29 Polymerase (Thermo Scientific) as previously described^17^. Firstly, a 20 μL reaction system consisting of 14 μL eccDNA (PSD-digested product), 4 μL 10 × phi29 reaction buffer and 2 μL Exo-resistant Random Primer (Invitrogen) was denatured at 95 °C for 5 min and ramped down to 4 °C at a ramping rate of – 5 °C/min. Then an additional mixture containing 4 μL dNTP (10 mM each), 0.8 μL DTT (100 mM), 1 μL phi29 polymerase (Thermo Scientific) and 14.2 μL ddH2O was added into the reaction system and incubated at 30°C continuously for 72 h. 1 μL RCA product of each sample was tested by agarose gel electrophoresis to confirm the amplification effect. Subsequently, the RCA product was purified using the VAHTS DNA Clean Beads (Vazyme) and eluted in 80 μL nuclease-free water. The concentration of the resulting RCA product was quantified by Qubit 4.0.

### Library preparation and sequencing

For the library construction for next-generation sequencing, 1 μg RCA product from each sample was fragmented into 300–400 bp fragments using sonication with Covaris LE220. The fragmented DNA was subsequently recovered and used to construct the sequencing library with the MGIEasy DNA Library Preparation Kit from MGI-BGI in China. The quality and length distribution of the libraries were evaluated using the Bioanalyzer 2100 from Agilent. Subsequently, the samples’ libraries were sequenced on the MGISeq-2000 platform (BGI) in paired-end mode, generating 150-base pair reads (PE150).

### Data pre-processing and alignment

Adapters or low-quality raw reads were trimmed using Trim Galore (v0.5.0) software (https://www.bioinformatics.babraham.ac.uk/projects/trim_galore) and Cutadapt (v1.18)^18^ with the following parameters: “--paired -q 28--fastqc--gzip”. The quality of processed clean data was evaluated using MultiQC (V1.15)^19^ and Qualimap (v2.3)^20^ with their default parameters. BWA-MEM (v 0.7.17)^21^ with default parameters was used to align the high-quality clean reads to the mouse reference genome (GRCm38/mm10), and the BAM files were sorted by Samtools^22^. Additionally, the aligned BAM files were sorted by sequence name using Samtools for further analysis.

### Identification of eccDNA by Cricle-Map

The Circle-Map (v1.1.4) software (https://github.com/iprada/Circle-Map) was used to extract circular reads from the sorted BAM files and identify eccDNAs based on the read coordinates in BAM files using default parameters. To improve the accuracy of eccDNA detection, multiple filtering steps were applied with the following specific criteria: (1) Split reads ≥ 2, (2) Circle score ≥ 200, (3) Coverage continuity ≤ 0.9, and (4) The standard deviation of coverage is smaller than the mean coverage across the entire eccDNA region.

### Annotation and analysis of eccDNA

The GFF3 annotation file of genome features was obtained from GENCODE version M23 (https://ftp.ebi.ac.uk/pub/databases/gencode/Gencode_mouse/release_M23/gencode.vM23.annotation.gff3.gz). The annotation data of candidate cis-Regulatory Elements (cCREs) was download from the ENCODE Project (https://www.encodeproject.org/). The data for repetitive DNA annotation was obtained from RepeatMasker open-4.0.5 (http://repeatmasker.org)^23^. To calculate the count of eccDNA mapped to the specific elements, we employed the multicov and groupby functions provided by BedTools^24^ with default parameters.

## Data Records

All the FASTQ files generated in this study by Circle-seq have been submitted and deposited in the NCBI Sequence Read Archive (SRA) with the BioProject accession number (PRJNA1012841)^25^. Additionally, the bed files containing information of eccDNA and the corresponding annotation data, as well as the detected results of eccDNA based on the mm39 genome, have also been uploaded to Figshare (10.6084/m9.figshare.24086121.v4)^26^.

## Technical Validation

### Quality check of the purified eccDNA samples

**Fig. 4:**
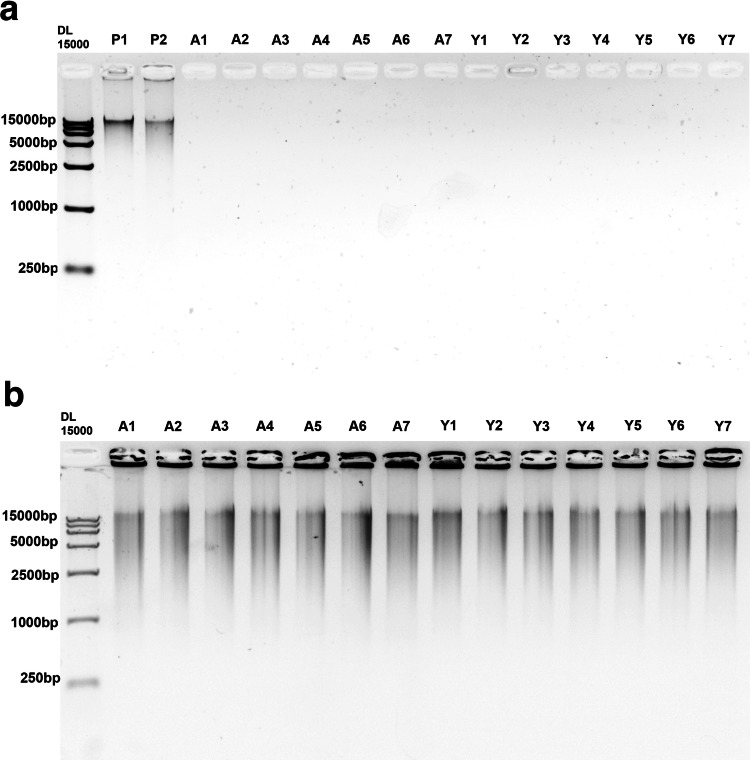
Validation of the purification of mouse brain eccDNA. (**a**) The results of agarose gel electrophoresis demonstrated that no bands indicative of linear DNA was detected in the samples obtained from mouse brain tissue, except for the positive control (P1 and P2). (**b**) The results of agarose gel electrophoresis consistently exhibited bands corresponding to the amplified products of eccDNA after rolling circle amplification.

To verify if linear DNA from chromosomes was completely digested, we examined the status of DNA on a 0.8% agarose gel. The results indicated that, except for two positive controls, no DNA content was detected in the remaining samples. (Fig. 4a). Subsequently, Rolling Circle Amplification was performed on the eccDNA and DNA detection was carried out again using a 0.8% agarose gel. The outcomes showed the existence of amplified products of eccDNA in all samples (Fig. 4b).

### Quality control of raw reads and sample statistics

**Fig. 5:**
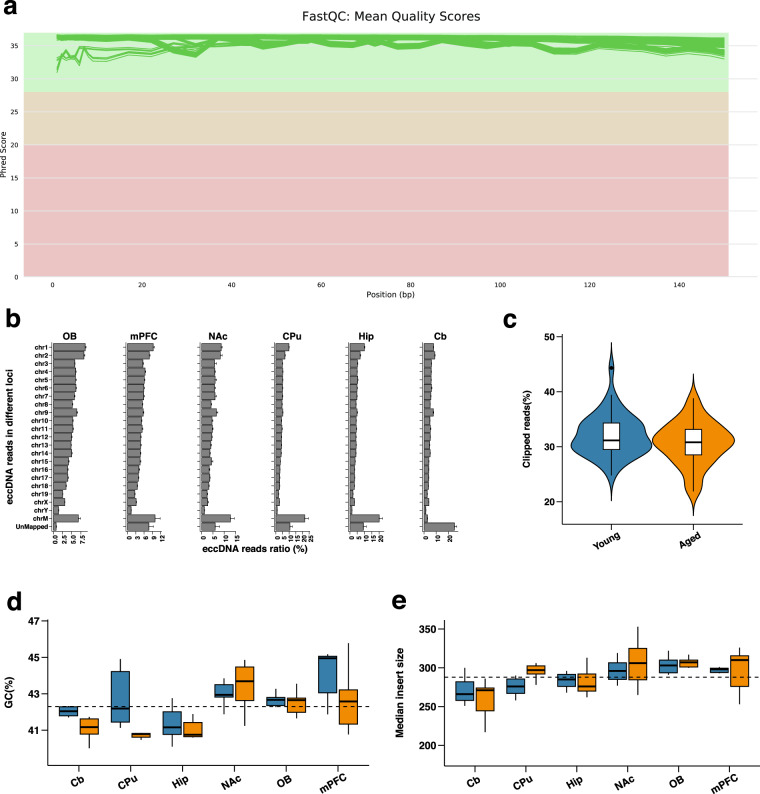
Quality control of Circle-seq data. (**a**) The line plot from FastQC demonstrates consistent high Phred Scores across the Circle-seq dataset in all samples. (**b**) Mapped ratios of eccDNA data across different chromosomes in six brain structure sequencing reads of mice. (**c**) Violin plot demonstrates the percentage of soft-clipped reads for both groups. (**d,e**) Box plot demonstrates the GC content and median insert size of the two groups separately. (Blue: young group and Orange: aged group).

We performed quality control on the Circle-seq data (Fig. 1b) and generated a series of QC metrics for Circle-seq (Supplementary Table 2). The overall quality of the Circle-seq dataset was deemed satisfactory at the levels of raw and mapped data in several features: (1) the Phred quality scores consistently exhibited a high level of quality in all samples (Fig. 5a); (2) the average ratio of mapping for all samples was 90.1% (Fig. 5b); (3) the average soft-clipped rate of 30.64% was determined for the aged group, while the young group had an average soft-clipped rate of 32.07% (Fig. 5c and Supplementary Table 3). This indicates the presence of a significant number of “split-reads” in the samples, which is also one of the characteristics of eccDNA; (4) the average GC content was approximately 41.88% for the aged group and 42.72% for the young group (Fig. 5d); (5) the average insert size (median length of the DNA fragment in the library) of each group ranged from 259 to 306 bp (Fig. 5e).

## Usage Notes

The Circle-seq data processing pipeline, which includes raw data filtering, reads alignment, eccDNA identification and genomic annotation, was executed on a Linux operating system. All the source codes in R and Python, along with the optimized parameters used for downstream data analyses and visualization, are available online for access.

### Supplementary information

Table S3

Table S1

Table S2

## Data Availability

The codes used to analyze the data in this study are available in the GitHub repository at the following URL: (https://github.com/XiaoningHong/MouseBrain_ScientificData).
